# Population‐level predictors of changes in success rates of smoking quit attempts in England: a time series analysis

**DOI:** 10.1111/add.14837

**Published:** 2019-12-01

**Authors:** Emma Beard, Sarah E. Jackson, Robert West, Mirte A. G. Kuipers, Jamie Brown

**Affiliations:** ^1^ Department of Behavioural Science and Health University College London London UK; ^2^ Department of Public Health, Amsterdam Public Health Research Institute, Amsterdam UMC University of Amsterdam Amsterdam the Netherlands

**Keywords:** ARIMAX, e‐cigarettes, mass media, NRT, quit success, time series, tobacco

## Abstract

**Aims:**

To quantify associations between the success of smoking quit attempts and factors that have varied throughout 2007–2018 at a population level.

**Design:**

time series analysis using Autoregressive Integrated Moving Average with Exogeneous Input (ARIMAX) modelling.

**Setting and Participants:**

Data were aggregated from 54 847 past‐year smokers taking part in the Smoking Toolkit Study which involves monthly repeated cross‐sectional household surveys of individuals aged 16+ in England.

**Measurements:**

The input series were: (1) attempts at smoking reduction using (a) e‐cigarettes and (b) nicotine replacement therapy (NRT); (2) use during a quit attempt of (a) e‐cigarettes, (b) NRT over‐the‐counter, (c) medication on prescription and (d) face‐to‐face behavioural support; (3) use of roll‐your‐own tobacco; (4) prevalence of (a) smoking and (b) non‐daily smoking; (5) tobacco control mass media expenditure; (6) expenditure on smoking; (7) smoker characteristics in the form of (a) high motivation to quit, (b) average age, (c) socio‐economic status and (d) cigarette consumption; (8) implementation of tobacco control policies; and (9) quit attempt rate.

**Findings:**

The licensing of NRT for harm reduction was associated with a 0.641% [95% confidence interval (CI) = 0.073–1.209, *P* = 0.027] increase in the mean point prevalence of the success rate of quit attempts. For every 1% increase in the mean point prevalence of e‐cigarette use and use of prescription medication during a quit attempt, the mean point prevalence of successful quit attempts increased by 0.106% (95% CI = 0.011–0.201, *P* = 0.029) and 0.143% (95% CI = 0.009–0.279, *P* = 0.038), respectively. For every 1% increase in the mean expenditure on tobacco control mass media, the mean point prevalence of successful quit attempts increased by 0.046% (95% CI = 0.001–0.092, *P* = 0.046). Other associations were not statistically significant.

**Conclusion:**

In England between 2007 and 2018, licensing of nicotine replacement therapy for use in harm reduction, greater use of e‐cigarettes and prescription medications during a quit attempt and higher expenditure on tobacco control mass media were all associated with higher success rates of quit attempts.

## Introduction

Globally the age‐standardized prevalence of tobacco smoking has decreased since the start of the 21st century from 26.9 to 20.2%, with a projected prevalence in 2025 of 15.5% 1. England has comprehensive tobacco control and one of the lowest smoking prevalences in the world at approximately 14.4% 2, 3. The Government Tobacco Control Plan for England aims to reduce smoking prevalence further to less than 12% by the end of 2022 4. Increasing the success rate of quit attempts will be important for achieving this target. The prevalence of successful quit attempts (defined as reporting still not smoking after a quit attempt made at some point within the past 12 months) has increased, varying around approximately 14% between 2007 and 2011 and approximately 17.5% thereafter (http://www.smokinginengland.info) 5. In order to inform and evaluate policy decisions to maintain or accelerate this increasing trend, it is important to track the success rate of quit attempts over time and identify factors that are associated with successful quit attempts at a population level. Thus, this paper used population‐level time series data on the success rate of quit attempts among past‐year smokers in England between 2007 and 2018 to quantify associations between the success of quit attempts and factors that have varied across the period at a population level and are known or hypothesized to influence quit attempts.

Given that a large proportion of smokers use e‐cigarettes and nicotine replacement therapy (NRT) to support harm reduction attempts—approximately 12.5 and 6.5%, in 2018 respectively—it is important to assess this influence at a population level 6. Survey studies and clinical trials suggest that NRT use for harm reduction increases smokers’ propensity to quit and the success of those quit attempts 7, 8. Even if this is true at an individual level, opponents of tobacco harm reduction are concerned that increased use of harm reduction approaches may make tobacco appear to be lower risk and lead people who otherwise would be using no tobacco or nicotine products to adopt the new product.

The success rate of quit attempts is likely to be influenced by prevalence of use of e‐cigarettes and other smoking cessation aids during a quit attempt. We recently showed, using population time series data, that part of the increase in prevalence of quit success in England over recent years may have been a consequence of the rise in use of e‐cigarettes during quit attempts 5. This is supported by findings from three randomized controlled trials (RCTs) which have shown that e‐cigarettes help smokers to stop smoking in the long term compared with placebo e‐cigarettes and NRT 9, 10. It is important to assess whether this association holds when accounting for other population‐level factors. We are unaware of any population‐level time series analyses that have assessed the effectiveness of prescription medication, but there is strong evidence from multiple RCTs for the efficacy of NRT, varenicline and bupropion with at least minimal professional support and face‐to‐face behavioural support 11, 12. In contrast, smokers who buy NRT over‐the‐counter with no behavioural support appear to have similar odds of success in stopping as those who stop without any aid 13.

The prevalence of successful quit attempts may also be affected by the overall prevalence of attempts to stop. It is possible that a rising quit attempt rate does not translate into improved quit success rates. A reduction in quit success may occur if, for example, triggering more people to try to stop meant that the pool of those trying to stop were less motivated or more dependent. The prevalence of roll‐your‐own tobacco use as a function of all cigarette use may play a role. Also referred to as ‘hand‐rolled’ cigarettes or ‘handmade’ cigarettes, roll‐your‐own consumption is on the increase in many countries 14. In the United Kingdom, it rose from 27% in 2007 to 33% in 2010 15. Although some studies have shown that the availability and use of cheap tobacco (including roll‐your‐own) is associated with lower success rates, other studies have found no such effects 16, 17, 18, 19.

The overall prevalence of smoking may influence success rates. It has been argued that as smoking prevalence declines, the remaining cohort of smokers includes disproportionate representation from groups of people who are more dependent and less able to quit successfully 20, 21. Although, more recently, the opposite argument of a softening has been put forward, due to dynamic changes in age and dependency levels of the smoking population 22. The proportion of non‐daily smokers as a function of all smokers may also have an impact on the prevalence of successful quit attempts, given that non‐daily smokers often struggle to quit smoking and are less likely than daily smokers to seek and receive smoking cessation treatment 23, 24. Although smoking prevalence has been gradually decreasing in high‐income countries, non‐daily smoking (also referred to as occasional smoking) appears to have become more prevalent 25, 26, 27.

Characteristics of smokers have changed since 2007, and this may account for changes in the success rate of quit attempts 28. For example, it appears that nicotine dependence has decreased among the remaining cohort of smokers in England 28, while motivation to quit has varied over time 29. Lower nicotine dependence has been shown to predict greater quit success 30. Although motivational factors predict the initiation of a quit attempt, there is little evidence at the individual level that these are associated with the success of quit attempts 30. Studies have also shown an increase in the age of smokers over time, but little change in the socio‐economic characteristics of smokers 28. This is likely to be partially attributable to reduced uptake in younger age groups, and highlights the lack of progress that has been made in reducing social inequalities. Socio‐economic status is a strong predictor of quitting activity 31, 32. Although smokers in more deprived socio‐economic groups are just as likely as those in more advantaged groups to try to stop, those in the most deprived group are half as likely to succeed 33, 34.

The first comprehensive tobacco control plan for Great Britain was published in 1998 and represented a substantial change in governmental approaches to tobacco control. It set the scene for a range of policies that have been enacted in subsequent years, including bans on tobacco marketing, a ban on smoking in indoor public areas, introduction of graphic health warnings on packs and increasing the legal age of sale 35. There is substantial evidence for the association between these population‐level tobacco control policies and quitting activities. The introduction of a smoking ban in July 2007 was associated with a significant temporary increase in the percentage of smokers attempting to stop 36, and the change in the minimum age of sale of cigarettes in October 2007 resulted in a greater fall in prevalence in 16–17 year‐olds 37. Pictorial health warnings on product packaging introduced in October 2008 have been shown to promote smoking cessation 38. An evaluation of the partial tobacco point‐of‐sale display ban introduced in England in April 2012 (only for large retail shops, with smaller shops requiring implementation by April 2015) found evidence for a decline in smoking prevalence 39. There have also been several other policies, including licensing of NRT for harm reduction in December 2009 40, the move in commissioning of stop smoking services to local authorities in April 2013 41 and the publication of National Institute for Health and Care Excellence (NICE) guidance on harm reduction in June 2013 42.

In England, tobacco control mass media campaigns have been run as part of a national tobacco control programme. Spending was almost completely suspended in 2010 and then reintroduced in 2011 at a much lower level. time series analyses have shown that such cuts on tobacco mass media expenditure are associated with a reduction in use of smoking cessation support 43, 44 and that higher monthly expenditure on tobacco control mass media campaigns in England is associated with a higher rate of quit success 45. It is important to assess if these findings hold when adjusting for other population level influences on quit success. Tax increases and a change in expenditure on smoking may also be associated with changes in quitting activity 46. This is in line with economic theory 47, which argues that tobacco price increases lead smokers to spend a higher share of their income on cigarettes and this increased expenditure on smoking provokes quit attempts and deters uptake 48, 49.

Thus, this paper aimed to examine the extent to which trends in the success of attempts to quit smoking in England since 2007 can be explained by a range of population‐level factors. Specifically, we are interested in the association of prevalence of the success of attempts to quit smoking in the past year with: (1) prevalence of e‐cigarettes for smoking reduction; (2) prevalence of NRT for smoking reduction; (3) prevalence of e‐cigarette use during a quit attempt; (4) prevalence of prescription medication use during a quit attempt; (5) prevalence of over‐the‐counter NRT use during a quit attempt; (6) prevalence of face‐to‐face behavioural support during a quit attempt; (7) prevalence of non‐daily smoking; (8) prevalence of roll‐your‐own tobacco; (9) mass media expenditure; (10) expenditure on tobacco; (11) average age of smokers; (12) prevalence of smokers in lower social grades (as a marker of socio‐economic status); (13) average cigarette consumption per day (as a marker of nicotine dependence); (14) prevalence of smokers with high motivation to quit; (15) population policies; (16) smoking prevalence; and (17) prevalence of quit attempts.

## Methods

### Design

Data on tobacco control mass media expenditure were obtained from Public Health England. Data for all other variables were used from the Smoking Toolkit Study (STS) (http://www.smokinginengland.info) between January 2007 and September 2018. The STS is a monthly survey of a representative sample of the population in England aged 16+ 50, which collects data on smoking patterns among smokers and recent ex‐smokers. The STS involves monthly household surveys using a random location sampling design, with initial random selection of grouped output areas (containing 300 households), stratified by ACORN (socio‐demographic) characteristics (http://www.caci.co.uk/acron/acornmap.asp) and region. Interviewers then choose which houses within these areas are most likely to fulfil their quotas and conduct face‐to‐face computer‐assisted interviews with one member per household. Participants from the STS appear to be representative of the population in England, having similar socio‐demographic composition to other large national surveys, such as the Health Survey for England 50.

### Measures

#### Outcome variable

Past‐year smokers were asked: ‘How many serious attempts to stop smoking have you made in the last 12 months? By serious attempt I mean you decided that you would try to make sure you never smoked again. Please include any attempt that you are currently making and please include any successful attempt made within the last year.’ Past‐year smokers who had reported making a quit attempt were then asked: ‘How long did your most recent serious quit attempt last before you went back to smoking? (a) still not smoking; (b) less than a day; (c) less a week; (d) more than 1 week and up to a month; (e) more than 1 month and up to 2 months; (f) more than 2 months and up to 3 months; (g) more than 3 months and up to 6 months; and (h) more than 6 months and up to a year.’ The success rate in each month was calculated as the number of respondents reporting that they were still not smoking at the time of the survey divided by the number reporting having made a quit attempt.

#### Explanatory variables

Participants were asked: ‘Which of the following best applies to you? (a) I smoke cigarettes (including hand‐rolled) every day; (b) I smoke cigarettes (including hand‐rolled), but not every day; (c) I do not smoke cigarettes at all, but I do smoke tobacco of some kind (e.g. pipe or cigar); (d) I have stopped smoking completely in the last year; (e) I stopped smoking completely more than a year ago; (f) I have never been a smoker (i.e. smoked for a year or more)’. Past‐year smoking prevalence in each month was calculated as the proportion of respondents who reported (a), (b) or (d). The prevalence of non‐daily smoking in each month was calculated as the proportion of current cigarette smokers (a or b) who reported (b). Current cigarette smokers were also asked how many hand‐rolled cigarettes they smoked per day. The prevalence of roll‐your‐own smoking in each month was calculated as the proportion of current cigarette smokers who reported that at least 50% of the cigarettes they smoked were roll‐your‐own cigarettes 19.

Current cigarette smokers were asked the following question: ‘Which, if any, of the following are you currently using to help you cut down the amount you smoke?’. This question had the following response options: nicotine gum, nicotine replacement lozenges/tablets, nicotine replacement inhaler, nicotine replacement nasal spray, nicotine patch, e‐cigarette, nicotine mouth spray, other, none. The prevalence of e‐cigarette use for smoking reduction in each month was calculated as the proportion who reported having used an e‐cigarette to cut down. The prevalence of NRT use for cutting down in each month was calculated as the proportion who reported having used any form of NRT.

The use of smoking cessation treatments was assessed only for the most recent quit attempt. Past‐year smokers who reported having made at least one quit attempt in the past 12 months were asked: ‘Which, if any, of the following did you try to help you stop smoking during the most recent serious quit attempt? (1) NRT on prescription, (2) bupropion, (3) varenicline, (4) specialist behaviour support (i.e. one‐to‐one or group behavioural support delivered by a National Health Service (NHS) Stop Smoking Service); (5) e‐cigarettes; and (6) NRT bought over the counter)’. The prevalence of prescription medication use each month was calculated as the proportion of respondents who reported having used NRT on prescription, bupropion or varenicline. The prevalence of e‐cigarette use in a quit attempt each month was calculated as the proportion of respondents who reported having used e‐cigarettes as an aid. The prevalence of use of NRT over the counter and face‐to‐face behavioural support was calculated each month as the proportion of respondents who reported having used each of these, respectively.

The prevalence of quit attempts in each month was calculated as the number of respondents who reported having made one or more quit attempt in the past 12 months divided by the number of past‐year smokers.

Motivation to quit was assessed using the Motivation to Stop Scale (MTSS) 51. Smokers were asked: ‘Which of the following describes you?’. The response categories were: (1) ‘I don't want to stop smoking’; (2) ‘I think I should stop smoking but don't really want to’; (3) ‘I want to stop smoking but haven't thought about when’; (4) ‘I REALLY want to stop smoking but I don't know when I will’; (5) ‘I want to stop smoking and hope to soon’; (6) ‘I REALLY want to stop smoking and intend to in the next 3 months’; (7) ‘I REALLY want to stop smoking and intend to in the next month’. The prevalence of high motivation to quit in each month was calculated as the proportion of smokers reporting that they intended to stop smoking within the next three months, i.e. response (6) or (7).

Cigarette consumption was measured by asking current smokers: ‘How many cigarettes per day do you usually smoke?’. We analysed the mean number reported by smokers in each monthly survey.

Past‐year smokers’ socio‐economic status was assessed by social grade measured using the British National Readership Survey (NRS) Social Grade Classification Tool 50: AB (higher managerial, administrative or professional), C1 (supervisory or clerical and junior managerial, administrative or professional), C2 (skilled manual workers), D (semi‐skilled and unskilled manual workers) and E (casual or lowest‐grade workers, pensioners and others who depend on the welfare state for their income). The prevalence of smokers in lower social grades was calculated as the proportion of past‐year smokers who reported being in C2, D and E. Past‐year smokers were also asked their age, with a mean estimated each month.

To assess expenditure on smoking, current smokers were asked: ‘On average about how much per week do you think you spend on cigarettes or tobacco?’ and to report the number of cigarettes they smoked per day (including hand‐rolled). Smokers’ average expenditure of smoking (in £/week) was derived from the following liberal assumptions for upper and lower estimates of plausible levels of consumption and expenditure per week: (1) smokers smoke a maximum of 560 cigarettes per week; (2) spending does not exceed £280 per week; and (3) single cigarettes expenditure between £0.05 and £1 52. Expenditure of smoking was adjusted for inflation using Consumer Prices Index (CPI) of all items from the Office for National Statistics.

Monthly tobacco mass media expenditure (in million £) was obtained from Public Health England. Total spending on campaigns was calculated for each month and included spending on ‘Smokefree’ campaigns, Stoptober campaigns and Health Harms campaigns. Spend included TV, radio, print, cinema and on‐line advertisements.

The following tobacco control policies were assessed using a temporary pulse effect (i.e. an effect lasting only for the month in which the policy was implemented), in which a dummy variable was coded 0 before and after the policy was introduced and 1 during the month the policy was introduced: (1) the introduction of a smoking ban in July 2007 36; (2) change in the minimum age of sale of cigarettes in October 2007 37; (3) pictorial health warnings on product packaging introduced in October 2008 38; (4) licensing of NRT for harm reduction in December 2009 40; (5) point‐of‐sale display ban introduced in England in April 2012 39; (6) the move in commissioning of stop smoking services to local authorities in April 2013 41; and (7) the publication of NICE guidance on harm reduction in June 2013 42.

### Analysis

The analysis plan and data set were pre‐registered on the Open Science Framework (https://osf.io/p8mwt/). All data were analysed in R 53. Data were aggregated monthly and weighted to match the population in England 50. Autoregressive Integrated Moving Average with Exogeneous Input (ARIMAX) analysis was used to estimate the effect of the explanatory variables on the outcome 54, 55, 56. ARIMAX is an extension of autoregressive integrated moving average analysis (ARIMA) which can accommodate both binary and continuous covariates. ARIMAX produces forecasts based upon prior values in the time series [autoregressive (AR) terms] and the errors made by previous predictions [moving average (MA) terms]. Standard recommended procedures were used to select the ARIMAX models 54, 57. Unadjusted (bivariate) models and a fully adjusted model (all variables included) are reported. Both unstandardized and standardized coefficients are provided. Bayes factors were derived using an online calculator for the final best fitting adjusted model 58 to disentangle whether there was evidence for the null hypothesis of no effect (Bayes factor < 1/3), insensitive data (Bayes factor between 1/3 and 3) or evidence for the alternative hypothesis of an effect (Bayes factor greater than 3). See Supporting information, File S1 for full details of the analysis. In all cases the best‐fitting model was a seasonal ARIMAX (0,1,1) (1,0,0), i.e. one order of non‐seasonal differencing, one order of non‐seasonal moving average autocorrelation [MA (1)] and one order of seasonal autoregressive autocorrelation [AR (1)]. Both the seasonal and non‐seasonal models [i.e. excluding seasonal AR (1)] are reported.

## Results

Data were collected from 248 897 respondents. Of these, 22.03% [95% confidence interval (CI) = 21.87–22.20; *n* = 54 847] were past year‐smokers and 20.02% (95% CI = 19.87–20.18; *n* = 49 838) were current smokers. Table 1 shows the mean, standard deviation and 95% CI of the time series for prevalence of quit success and the predictor time series of interest. The Supporting information, Figs S1, S2 and S3 shows the plotted time series. On average, 16.20% (95% CI = 15.45–16.95) of respondents reported a successful quit attempt, with an increase from approximately 14% between 2007 and 2011 to 18% thereafter.

**Table 1:** Descriptive statistics of the time series of prevalence of successful quit attempts over time and the predictor time series of interest.

	Mean	SD	95% confidence interval	
			Lower	Higher
Quit success	16.20	4.54	15.45	16.95
E‐cigarette use for SR	7.40	6.35	6.35	8.45
NRT use for SR	9.70	3.92	9.05	10.35
Non‐daily smokers	11.70	2.82	11.23	12.17
Roll‐your‐own smokers	40.00	7.43	38.77	41.23
Tobacco control mass media spend (£ millions)	0.50	0.48	0.42	0.58
Smokers’ expenditure on smoking	26.00	1.58	25.74	26.26
Lower social grade	59.50	2.79	59.04	59.96
Age	41.30	0.86	41.16	41.44
High motivation to quit	18.40	3.97	17.75	19.05
Cigarettes per day	12.10	1.06	11.92	12.28
Smokers	22.00	2.56	21.58	22.42
E‐cigarette use during a quit attempt	16.90	16.06	14.25	19.55
Prescription medication use during a quit attempt	14.30	5.83	13.34	15.26
NRT use OTC during a quit attempt	25.60	7.45	24.37	26.83
Face‐to‐face behavioural support during a quit attempt	4.20	2.30	3.82	4.58
Quit attempts	35.60	4.74	34.82	36.38

SR = smoking reduction; NRT = nicotine replacement therapy; OTC = over‐the‐counter; SD = standard deviation.

Table 2 presents the unadjusted ARIMAX analyses. A positive temporary impact on quit success was found for the increase in age of sale of tobacco and change in licensing of NRT for harm reduction purposes. A positive association was also found with e‐cigarette use during a quit attempt and use of prescription medication during a quit attempt, while a negative association was found with NRT use for smoking reduction.

**Table 2:** Results of the unadjusted (bivariate) Autoregressive Integrated Moving Average with Exogeneous Input (ARIMAX) models assessing the association between the variables of interest and prevalence of successful quit attempts.

	B No seasonal AR Seasonal AR	Lower CI	Upper CI	*P*	Β No seasonal AR Seasonal AR	
Partial point‐of‐sale ban	0.060	−0.456	0.577	0.819		ARIMAX (0,1,1)
	0.060	−0.456	0.577	0.819		No lag
Smoke‐free	0.178	−0.340	0.696	0.501		ARIMAX (0,1,1)
	0.178	−0.340	0.696	0.501		No lag
Increase in age of sale	0.513	0.003	1.023	0.049		ARIMAX (0,1,1)
	0.514	0.004	1.025	0.048		1 month
Pictorial health warnings	−0.247	−0.764	0.269	0.348		ARIMAX (0,1,1)
	−0.248	−0.764	0.269	0.347		No lag
Move of Stop Smoking Services to local authority control	0.079	−0.436	0.593	0.765		ARIMAX (0,1,1)
	0.079	−0.437	0.595	0.765		No lag
Licensing of NRT for harm reduction	0.522	0.014	1.029	0.044		ARIMAX (0,1,1)
	0.523	0.015	1.030	0.044		1 month
NICE guidance on harm reduction	0.254	−0.259	0.768	0.331		ARIMAX (0,1,1)
	0.254	−0.259	0.768	0.331		No lag
E‐cigarette use for SR	0.052	−0.003	0.108	0.062	0.059	ARIMAX (0,1,1)
	0.052	−0.003	0.108	0.062	0.059	No lag
Tobacco control mass media spend (£ millions)	0.046	−0.005	0.096	0.076	0.101	ARIMAX (0,1,1)
	0.047	−0.004	0.098	0.071	0.102	No lag
NRT use for SR	−0.203	−0.391	−0.01	0.034	−0.422	ARIMAX (0,1,2)
	−0.202	−0.389	6−0.015	0.034	−0.421	No lag
Roll‐your‐own smokers	0.506	−0.017	1.029	0.058	0.322	ARIMAX (0,1,1)
	0.515	−0.015	1.046	0.057	0.322	No lag
Non‐daily smokers	−0.700	−0.264	0.125	0.482	−0.035	ARIMAX (0,1,1)
	−0.700	−0.266	0.125	0.480	−0.036	No lag
Smokers’ expenditure on smoking	−0.458	−1.272	0.357	0.271	−0.093	ARIMAX (0,1,1)
	−0.461	−1.277	0.355	0.268	−0.096	No lag
Cigarettes per day	0.032	−0.899	0.963	0.946	0.009	ARIMAX (0,1,1)
	0.032	−0.901	0.966	0.946	0.010	No lag
Smokers	0.219	−0.452	0.889	0.523	0.017	ARIMAX (0,1,1)
	0.219	−0.452	0.889	0.523	0.018	No lag
High motivation to quit	0.021	−0.278	0.339	0.846	−0.020	ARIMAX (0,1,1)
	0.031	−0.279	0.340	0.846	−0.021	No lag
Age	−0.006	−2.385	2.372	0.996	−0.019	ARIMAX (0,1,1)
	−0.006	−2.385	2.372	0.996	−0.019	No lag
Lower social grade	0.140	−0.906	1.186	0.793	0.019	ARIMAX (0,1,1)
	0.140	−0.908	1.189	0.793	0.019	No lag
E‐cigarette use during a quit attempt	0.063	0.037	0.089	<0.001	0.377	ARIMAX (0,1,1)
	0.063	0.037	0.089	<0.001	0.376	No lag
Prescription medication use during a quit attempt	0.177	0.024	0.330	0.023	0.047	ARIMAX (0,1,1)
	0.177	0.025	0.330	0.023	0.047	No lag
NRT use OTC during a quit attempt	0.034	−0.197	0.266	0.770	−0.006	ARIMAX (0,1,1)
	0.035	−0.198	0.267	0.769	−0.006	No lag
Face‐to‐face behavioural support during a quit attempt	0.043	−0.031	0.116	0.254	0.064	ARIMAX (0,1,1)
	0.044	−0.031	0.118	0.248	0.066	No lag
Quit attempts	−0.240	−0.677	0.197	0.283	−0.024	ARIMAX (0,1,1)
	−0.246	−0.689	0.197	0.277	−0.025	No lag

SR = smoking reduction; NRT = nicotine replacement therapy; OTC = over‐the‐counter; AR = autoregressive correlation; CI = confidence interval; NICE = National Institute for Health and Care Excellence.

Table 3 presents the adjusted ARIMAX analyses. The licensing of NRT for harm reduction was associated with a 0.641% (95% CI = 0.073–1.209, *P* = 0.027) increase in the mean point prevalence of the success rate of quit attempts. For every 1% increase in the mean point prevalence of e‐cigarette use and use of prescription medication during a quit attempt, the mean point prevalence of successful quit attempts increased by 0.106% (95% CI = 0.011–0.201, *P* = 0.029) and 0.143% (95% CI = 0.009–0.279, *P* = 0.038), respectively. For every 1% increase in the mean expenditure on tobacco control mass media, the mean point prevalence of successful quit attempts increased by 0.046% (95% CI = 0.001–0.092, *P* = 0.046).

**Table 3:** Results of the adjusted Autoregressive Integrated Moving Average with Exogeneous Input (ARIMAX) models assessing the association between the variables of interest and prevalence of successful quit attempts.

	B No seasonal AR Seasonal AR	Lower CI	Upper CI	P	Β No seasonal AR Seasonal AR	Bayes factors
Partial point‐of‐sale ban	0.127	−0.357	0.611	0.607		1.09
	0.131	−0.354	0.616	0.596		1.10
Smoke‐free	0.188	−0.300	0.676	0.451		1.18
	0.192	−0.298	0.681	0.443		1.19
Increase in age of sale (1 month lag)	0.446	−0.049	0.941	0.077		1.71
	0.439	−0.060	0.937	0.084		1.67
Pictorial health warnings	−0.134	−0.610	0.343	0.583		1.10
	−0.132	−0.608	0.343	0.586		1.10
Move of Stop Smoking Services to local authority control	0.204	−0.284	0.692	0.412		1.21
	0.196	−0.296	0.688	0.435		1.19
Licensing of NRT for harm reduction (1 month lag)	0.634	0.068	1.200	0.028		1.87
	0.641	0.073	1.209	0.027		1.88
NICE guidance on harm reduction	0.219	−0.290	0.727	0.399		1.21
	0.208	−0.306	0.723	0.428		1.19
E‐cigarette use for SR	−0.040	−0.110	0.030	0.262	0.619	1.00
	−0.040	−0.110	0.030	0.259	0.672	1.00
Tobacco control mass media spend	0.048	0.003	0.092	0.036	0.057	3.64
	0.046	0.001	0.092	0.046	0.058	2.84
NRT use for SR	−0.175	−0.384	0.034	0.101	−0.607	2.38
	−0.177	−0.387	0.032	0.097	−0.662	2.41
Roll‐your‐own smokers	0.572	−0.019	1.163	0.058	0.086	1.65
	0.583	−0.016	1.182	0.056	0.082	1.64
Non‐daily smokers	−0.015	−0.223	0.193	0.885	−0.014	0.79
	−0.025	−0.249	0.198	0.823	−0.012	0.85
Smokers’ expenditure on smoking	0.140	−0.906	1.185	0.793	0.012	1.02
	0.127	−0.923	1.176	0.813	0.013	1.02
Cigarettes per day	0.063	−1.009	1.135	0.908	−0.008	1.00
	0.071	−1.003	1.145	0.897	−0.009	1.00
Smokers	0.530	−0.124	1.183	0.112	0.072	1.44
	0.529	−0.125	1.184	0.113	0.072	1.44
High motivation to quit	0.045	−0.265	0.355	0.777	−0.006	0.96
	0.043	−0.267	0.354	0.784	−0.005	0.95
Age	0.517	−1.921	2.955	0.678	0.01	1.03
	0.559	−1.899	3.017	0.656	0.01	1.03
Lower social grade	−0.224	−1.211	0.764	0.657	−0.005	1.05
	−0.247	−1.253	0.759	0.631	−0.004	1.06
E‐cigarette use during a quit attempt	0.105	0.010	0.200	0.030	0.197	5.69
	0.106	0.011	0.201	0.029	0.202	5.90
Prescription medication use during a quit attempt	0.144	0.009	0.279	0.037	0.099	4.78
	0.143	0.008	0.278	0.038	0.100	4.68
NRT use OTC during a quit attempt	0.096	−0.137	0.328	0.420	0.027	1.23
	0.102	−0.136	0.340	0.403	0.028	1.25
Face‐to‐face behavioural support during a quit attempt	0.041	−0.032	0.115	0.267	0.036	1.01
	0.043	−0.031	0.118	0.256	0.034	1.06
Quit attempts	−0.135	−0.553	0.282	0.525	−0.024	1.14
	−0.125	−0.551	0.301	0.565	−0.026	1.11

SR = smoking reduction; NRT = nicotine replacement therapy; OTC = over‐the‐counter; AR = autoregressive correlation; CI = confidence interval; NICE = National Institute for Health and Care Excellence.

Bayes factors for all other non‐significant findings suggested that the data were insensitive to detect an association (Bayes factor = 0.79–2.41) (see Table 3).

## Discussion

### Key findings

An increase in the prevalence of use of e‐cigarettes, use of prescription medication during a quit attempt and expenditure on tobacco mass media campaigns were each associated with a significantly higher prevalence of successful quit attempts among past‐year smokers in England. The introduction of the licensing of NRT for harm reduction also had a temporary positive impact on the prevalence of successful quit attempts. Bayes factors indicated that the data were insensitive to detect an association between the other variables and prevalence of successful quit attempts.

### Strengths and limitations

This is the first study, to our knowledge, that has assessed the association of a wide range of population‐level factors with successful quit attempts. The use of ARIMAX modelling is a strength because it accounts for underlying trends, seasonality and autocorrelation. ARIMAX also has added value over traditional regression models by using all the information in past data to estimate coefficients.

This study also has several limitations. First, the STS requires participants to recall certain key smoking‐related factors which could have introduced bias. However, this would only have had a significant effect on time series models if there was a change in the rate of under‐ or over‐reporting over time. Secondly, the findings may not generalize to other countries. England has a strong tobacco control climate, generally high motivation to quit among smokers and relatively liberal regulation of e‐cigarettes. In countries with weaker tobacco control, or stricter regulation of e‐cigarettes, different effects may be observed. Thirdly, although we are unaware of any other major population‐level interventions or other events during the study period, we cannot rule out residual confounding. Fourthly, caution should be taken when interpreting the null effects. Although this study was powered to detect relatively small associations, Bayes factors indicated that the presence of a *P*‐value greater than 0.05 in a number of instances was because the data were inconclusive as to whether an association exists, rather than there being evidence for no effect 58, 59. Fifthly, the failure to find an impact of several population‐level polices which have previously been evaluated as successful may reflect that we only modelled pulse and delayed pulse effects. It is possible that findings might be different if a more comprehensive evaluation had been undertaken; for example, a consideration of step‐level changes or permanent changes in quit rates. This was not possible with the number of different factors included in the models in the current study, but warrants further investigation. Sixthly, time series analyses are dependent on data variability. Thus, where there is little fluctuation, as was the case with lower socio‐economic status, associations may not be identifiable. Seventhly, although the STS has a measure of dependence, the Heaviness of Smoking Index (HSI), the low variability over time meant that it was not suitable for ARIMAX modelling. Cigarettes per day as a measure related to dependence was chosen instead. Finally, this paper only considered one component of stopping smoking, it will be important to assess if the predictors of quit attempts and not necessarily the success are similar.

### Interpretation of the findings

Previous studies have similarly found positive associations between tobacco control mass media spend and the success of quit attempts 45. One possible mechanism may be that tobacco control mass media campaigns sustain motivation to persist with a quit attempt by maintaining the salience of quitting or normalizing quitting 60. Although changes in high motivation to quit were unrelated to quit success in the current study, this measure assessed desire and intention to quit among people who were currently smoking, and motivation to persist among ex‐smokers was not assessed. Another mechanism may be that exposure to campaigns may encourage smokers to seek professional help. This is supported by evaluations in the United States and England which have identified positive associations with the use of quit‐lines and other aids 43, 61. These findings are also in line with evidence on the efficacy of e‐cigarettes from RCTs, and supports the view that e‐cigarette use in a quit attempt can aid smoking cessation 9, 10.

We also found, consistent with observational studies and RCTs, that the use of prescription medication was positively associated with quit success 11, 12. This is the first population‐level time series analysis, to our knowledge, that has assessed the real‐world effectiveness of prescription medication for smoking cessation while adjusting for a wide range of covariates. To maximize statistical power, we combined the prescription of NRT, bupropion and varenicline in our study. It would be useful to compare these medications with each other if sufficient samples are accumulated. However, use of bupropion and varenicline has declined substantially in recent years 62.

In 2009, the UK Medicines and Healthcare products Regulatory Agency expanded the marketing license for a number of NRT products to include smoking reduction without an intention to stop completely. This was following the accumulation of evidence that use of NRT for harm reduction increased smokers’ propensity to quit at a population level and in clinical trials 7, 8. Although our previous individual‐level analyses failed to find any association of this expansion with the success of quit attempts 63, our current study identified a positive impact. These conflicting results may be due to a lack of power in previous analyses to detect subtle changes or the failure to account for other population level policies and seasonal effects. Nonetheless, these findings are surprising, given that the licensing change did not incorporate any specific promotion to educate smokers about how to use NRT for harm reduction purposes. Although many NRT users reportedly read package inserts, smokers do not appear to do so carefully 64, 65.

### Implications

These findings have several policy implications. First, they suggest that tobacco control mass media campaigns have the potential to increase quit success, and therefore to reduce smoking prevalence, reduce life years lost to smoking and to have a positive return on investments. Thus, there is a need to encourage the UK government to increase their spend on mass media campaigns. There has been a considerable reduction in government investment during the last few years. In 2012/13 the national spend on mass media was approximately 8 million, which fell to £1.5 million in 2016–17 and £2 million in 2017/18 66. Secondly, they support the use of both e‐cigarettes and prescription medication for smoking cessation 6, 9, 10, 11, 67. Health‐care professionals should be encouraged to offer smoking cessation medication, including e‐cigarettes, to smokers attempting to quit. Thirdly, it would also be worth exploring some of the population level policies further, including the licensing of NRT for harm reduction, as a great deal of time and resources go into their development and delivery.

## Conclusion

Increases in the prevalence of e‐cigarette use and prescription medication use during a quit attempt, and expenditure on tobacco control mass media campaigns, was associated with a higher prevalence of successful quit attempts. Licensing of NRT for harm reduction also appeared to have a temporary positive impact on the prevalence of successful quit attempts.

## Declaration of interests

R.W. undertakes consultancy and research for and receives travel funds and hospitality from manufacturers of smoking cessation medications. E.B. and J.B. have received unrestricted research funding from Pfizer. M.K. and S.J. have no interests to declare.

## Supporting information


**Figure S1** Plotted time series of prevalence of successful quit attempts and a) prevalence of e‐cigarette use for smoking reduction; b) prevalence of NRT use for smoking reduction; c) tobacco control mass media spend; d) expenditure on smoking; e) prevalence of high motivation to quit; and f) prevalence of lower socio‐economic status.

**Figure S2** Plotted time series of prevalence of successful quit attempts and a) smoking prevalence; b) non‐daily smoking prevalence; c) average cigarette consumption per day; d) average age of smokers and e) prevalence of roll‐your‐own smokers.

**Figure S3** Plotted time series of prevalence of successful quit attempts and a) prevalence of use of over‐the‐counter NRT during a quit attempt; b) prevalence of prescription medication use during a quit attempt; c) prevalence of face‐to‐face behavioural support use during a quit attempt; d) prevalence of e‐cigarette use during a quit attempt and e) prevalence of quit attempts.

**Table S1** Descriptive statistics of the time series of prevalence of successful quit attempts over time and the predictor time series of interest with outlier replacement.
**Table S2** Results of the unadjusted ARIMAX models assessing the association between the variables of interest and prevalence of successful attempts to quit smoking with outlier replacement.
**Table S3** Results of the adjusted ARIMAX models assessing the association between the variables of interest and prevalence of successful quit attempts with outlier replacement.
