# Supplementary figures and images for: Population‐level predictors of changes in success rates of smoking quit attempts in England: a time series analysis
Source: Addiction. 2019 Dec 1;115(2):315–25. doi: 10.1111/add.14837 (PMC7004132; doi:10.1111/add.14837)

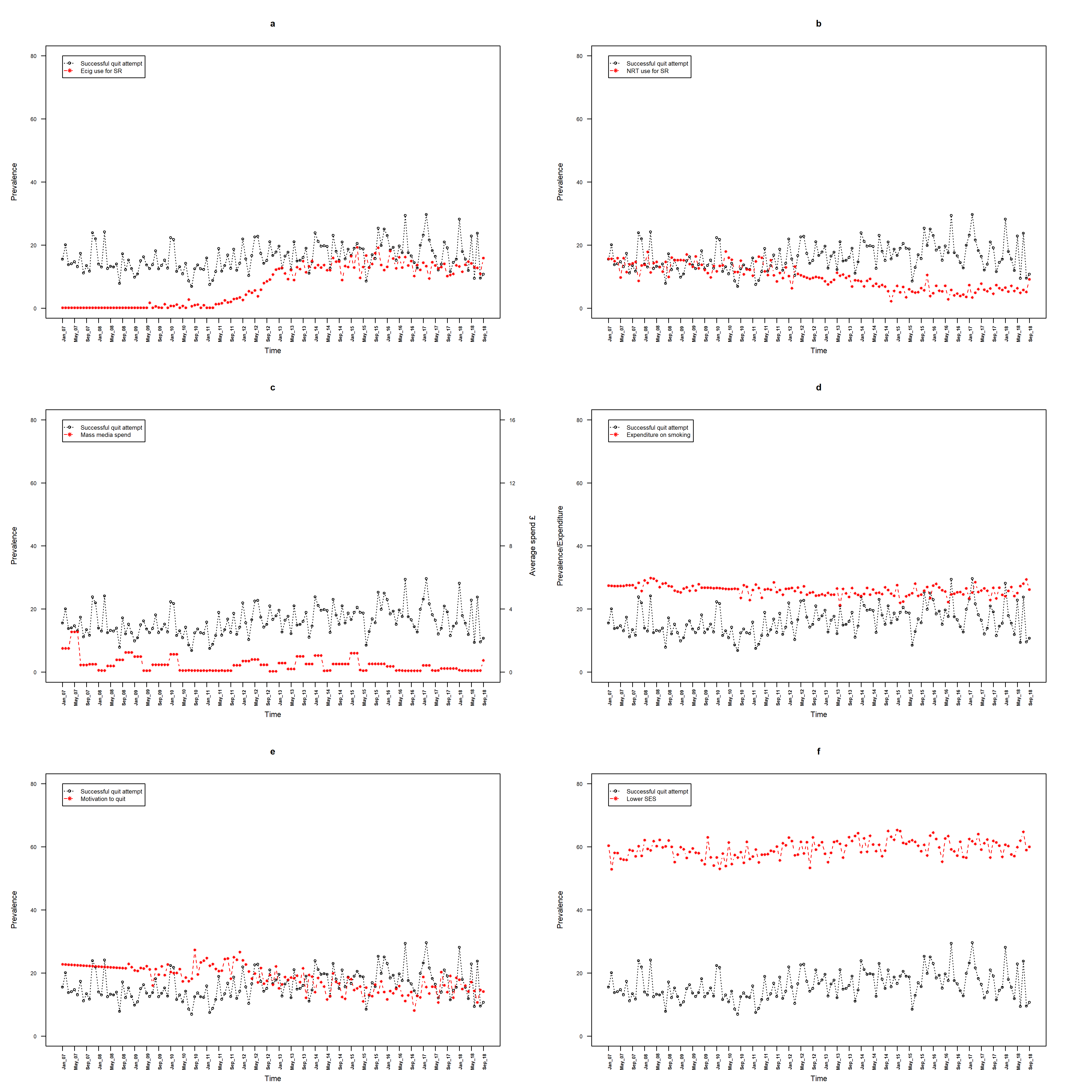

Supplement: Supplementary file 1 — Figure S1 Plotted time series of prevalence of successful quit attempts and a) prevalence of e‐cigarette use for smoking reduction; b) prevalence of NRT use for smoking reduction; c) tobacco control mass media spend; d) expenditure on smoking; e) prevalence of high motivation to quit; and f) prevalence of lower socio‐economic status. [file ADD-115-315-s001.tiff]

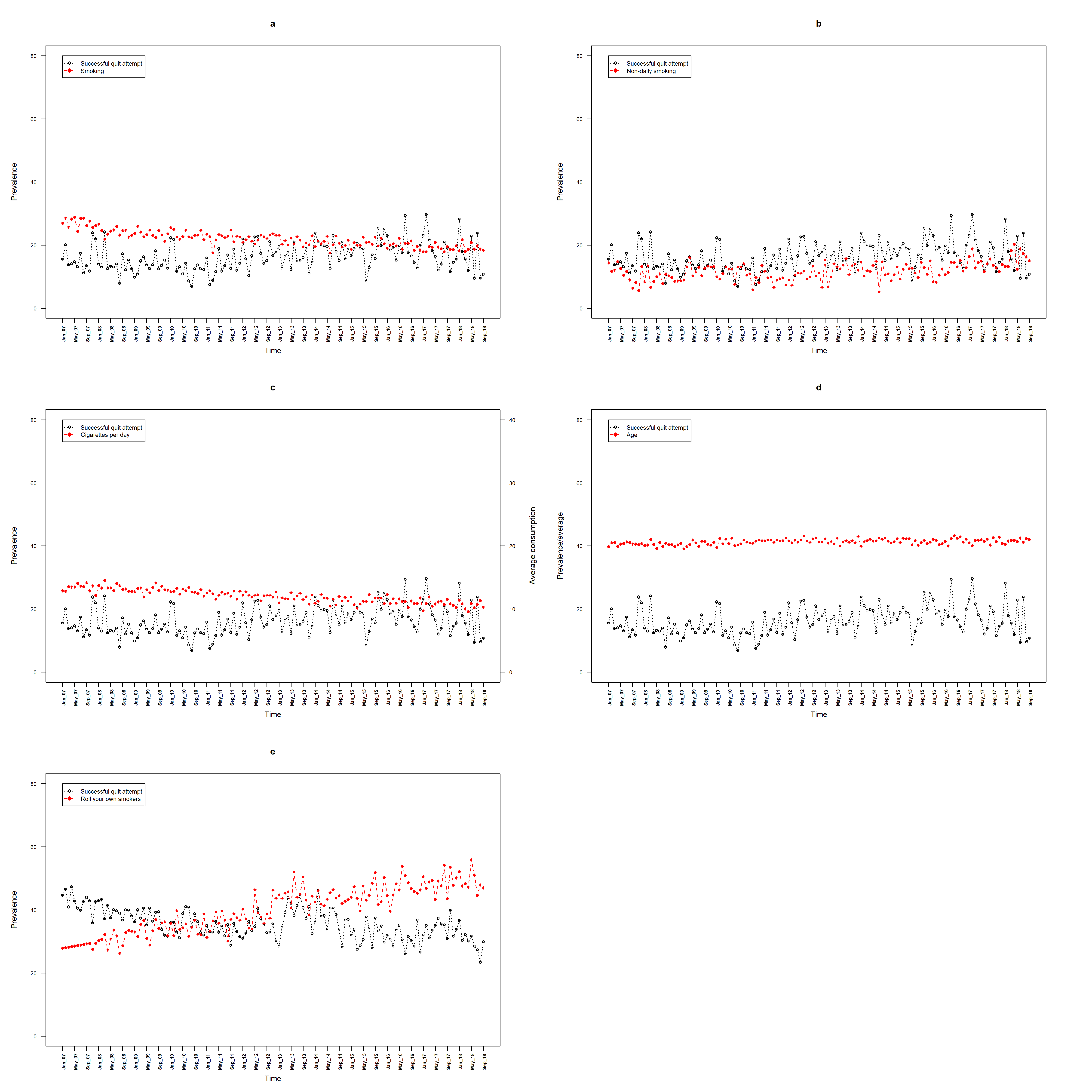

Supplement: Supplementary file 2 — Figure S2 Plotted time series of prevalence of successful quit attempts and a) smoking prevalence; b) non‐daily smoking prevalence; c) average cigarette consumption per day; d) average age of smokers and e) prevalence of roll‐your‐own smokers. [file ADD-115-315-s002.tiff]

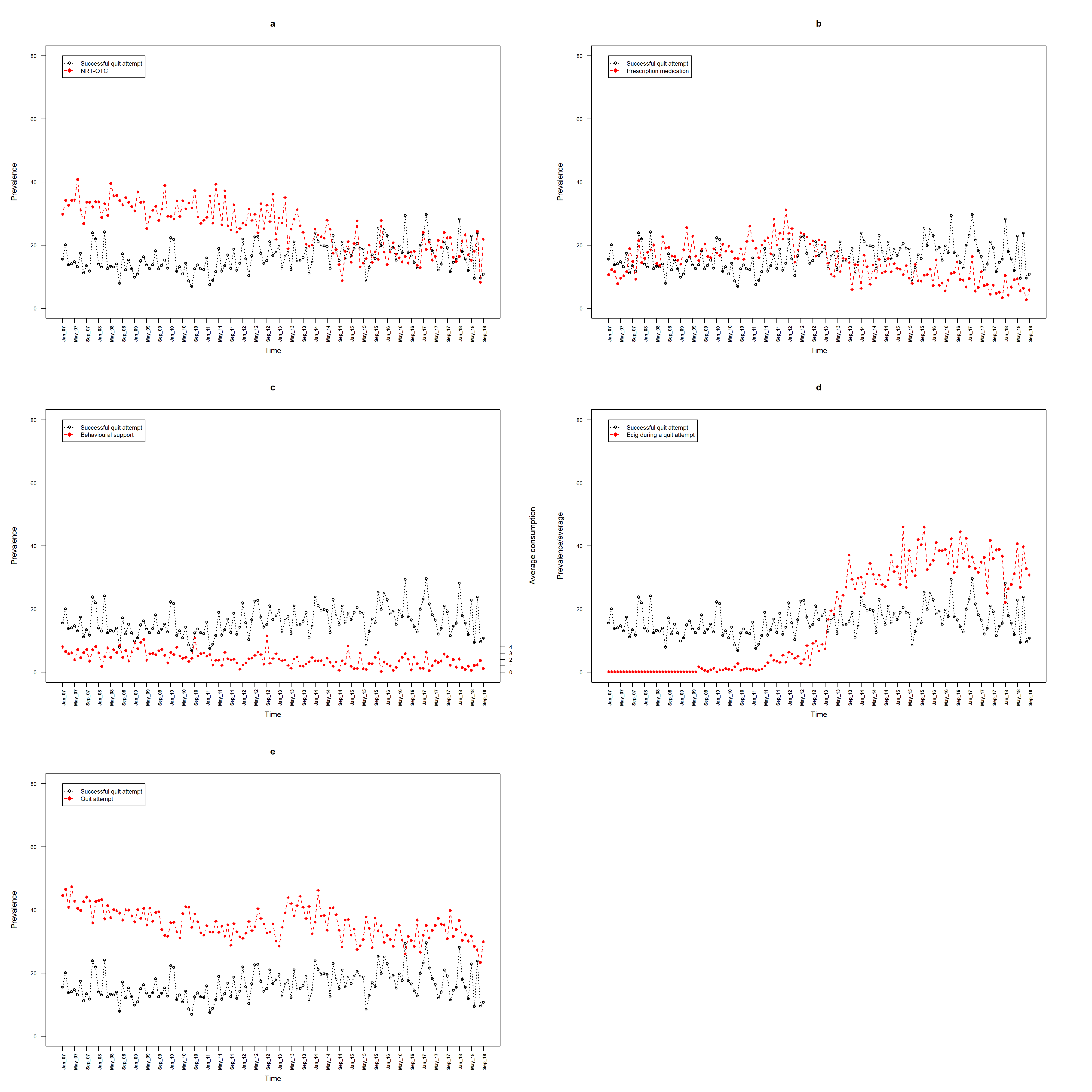

Supplement: Supplementary file 3 — Figure S3 Plotted time series of prevalence of successful quit attempts and a) prevalence of use of over‐the‐counter NRT during a quit attempt; b) prevalence of prescription medication use during a quit attempt; c) prevalence of face‐to‐face behavioural support use during a quit attempt; d) prevalence of e‐cigarette use during a quit attempt and e) prevalence of quit attempts. [file ADD-115-315-s003.tiff]
