# Supplementary material for: Population‐level predictors of changes in success rates of smoking quit attempts in England: a time series analysis
Source: Addiction. 2019 Dec 1;115(2):315–25. doi: 10.1111/add.14837 (PMC7004132; doi:10.1111/add.14837)
Supplement: Supplementary file 4 — Table S1 Descriptive statistics of the time series of prevalence of successful quit attempts over time and the predictor time series of interest with outlier replacement. Table S2 Results of the unadjusted ARIMAX models assessing the association between the variables of interest and prevalence of successful attempts to quit smoking with outlier replacement. Table S3 Results of the adjusted ARIMAX models assessing the association between the variables of interest and prevalence of successful quit attempts with outlier replacement. [file ADD-115-315-s004.docx]

***Analysis***

The analysis plan and data set were pre-registered on the Open Science Framework (<https://osf.io/p8mwt/>). All data were analysed in R (1). Data were aggregated monthly and weighted to match the population in England (2).

*Missing data*

Questions on roll-your-own cigarette use were not introduced until November 2007 and questions on motivation to quit were not introduced in November 2008. Questions on the cost of cigarettes were only collected between October 2007 and June 2009 and then from August 2010 onwards. Data were only available on the prevalence of use of e-cigarettes among smokers for cutting down from April 2011 – although use specifically during a recent quit attempt were available from July 2009. Thus, prevalence of e-cigarette use for cutting down among smokers between July 2009 and April 2011 were estimated from data on use during a quit attempt. Use of e-cigarettes for harm reduction among smokers between November 2006 and June 2009, cost of cigarettes, use of roll-you-own and motivation to quit were imputed using Kalman Smoothing for univariate time series data (3). See (4, 5) for a detailed introduction to Kalman filtering. For a year, alternate months (May-2012, Jul-2012, Sep-2012, Nov-2012, Jan-2013, Mar-2013) did not record data on e-cigarettes and NRT use among smokers for harm reduction. Data were imputed using the average of the month before and the month after.

Two waves of data were collected in March 2007 and March 2013. These waves were averaged. No data were collected in December 2008. Data were imputed using the average of the month before and the month after.

For a number of months, mass media spending was effectively zero and was imputed with random numbers generated from a truncated normal distribution with mean 0.1, SD 0.01 and bounds of 0 to infinity.

*Main analysis*

Autoregressive Integrated Moving Average with Exogeneous Input (ARIMAX) analysis was used to estimate the effect of the explanatory variables on the outcome (6-8). ARIMAX is an extension of autoregressive integrated moving average analysis (ARIMA) which can accommodate both binary and continuous covariates. ARIMAX produces forecasts based upon prior values in the time series (autoregressive [AR] terms) and the errors made by previous predictions (moving average [MA] terms). Unadjusted models and a fully adjusted model were reported. Both unstandardized and standardised coefficients were provided.

Standard recommended procedures were used to select the ARIMAX models (6, 9). First, each time series was assessed for outlying values which may bias the results using a procedure described in Chen and Liu (1993) (10). This involved fitting a loess curve and residuals identified as outliers if they were outside the range $\pm2\left( q_{0.9}-q_{0.1} \right),$ where the $q_{p}$ is the p-quartile of the residuals. Outliers were identified for e-cigarette use during a quit attempt. The outliers were identified in waves 81, 99, 100, 104, 106, 118, 127, 128, 133, 137 and 139. These were the values 37.1, 46.04, 26.89, 42.02, 46.01, 44.48, 25.04, 41.81, 22.22, 40.7, and 39.71, and the suggested replacements were: 28.195, 31.380, 34.960, 35.485, 36.455, 34.735, 34.785, 36.260, 36.160, 31.585, 29.045 and 29.850, respectively. Outliers were also identified for mass media expenditure. The outliers were identified in waves 4, 5 and 6. These were the values 2.55, 2.55 and 2.55 respectively, and the suggested replacements were 1.24, 0.98 and 0.72, respectively. These outliers were not deemed to be aberrant but real true values of prevalence and expenditure. Following standard recommend procedures, as the outliers did not affect the results (see Supplementary Tables 1-3 below) the main analyses are presented without outlier replacement (11).

Secondly, we assessed the assumption of weak exogeneity using the default order of lags of 1 up to a lag of 4 (i.e., that there is no reverse causal pathway such that Y can depend on the lagged values of X, but not vice versa) using the Granger Causality test. There was no evidence that the assumption was violated for any of the models (at α = 0.001). Secondly, we created plots of the differenced data and ran unit root tests (i.e. Osborn-Chui-Smith-Birchenhall test and Kwiatkowski, Phillips, Schmidt, and Shin (KPSS) test) to determine the number of seasonal and non-seasonal differences required for the time series to be made stationary (12, 13).

Thirdly, to identify the most appropriate transfer function for the continuous explanatory variables, up to 4 lags (i.e. to identify the manner in which past values of the electronic cigarette time series are used to forecast future values of the outcome), the sample cross-correlation function was checked for each ARIMAX model, with pre-whitened data (8). Pre-whitening removes autocorrelation in the input series that may cause spurious cross-correlation effects and therefore aids interpretation. Additional checks were also be run by comparing univariate ARIMAX models with variations for the transfer function using the AIC. For the dummy coded policy variables, lags were assessed by deriving shifted policy variables with lags of 1, 2, 3 and 4 months and models compared using the AIC. Lags greater than 4 (representing more than four months) were deemed to be unlikely, with policies expected to have an almost immediate effect.

Fourthly, to determine the initial values of the AR and MA terms for the baseline models, the autocorrelation function (ACF) and partial autocorrelation function (PACF) were assessed. Additional models with various fitted AR and MA terms were then compared to this baseline model using the Akaike information criterion (AIC). According to the Box-Jenkins method, in ARIMA (p, d, q) the value of p and q should be 2 or less or the total number of parameters should be less than 3 (6). Therefore, we only checked ARIMAX models for p and q values of 3 or less. The models with lower AIC values were selected. Fifthly, the ACF for the residuals of the best fitting models were checked for additional correlation (thus the need for additional MA/AR seasonal or non-seasonal terms) and the coefficients of the correlation terms assessed for significance and whether they fell within the bounds of stationarity and invertibility (14, 15).

Finally, the Ljung-Box test for white noise and the ACF for the residuals of the best fitting models were checked for additional correlation (thus the need for additional MA/AR seasonal or non-seasonal terms) and the coefficients of the correlation terms assessed for significance and whether they fell within the bounds of stationarity and invertibility (14, 15). The Ljung-Box test is a portmanteau test which instead of testing randomness at each distinct lag, it tests the "overall" randomness based on a number of lags. The residuals for all of the models were not free of serial correlation (16). This was amended with the addition of a seasonal AR term i.e. a seasonal ARIMAX(0,1,1)(1,0,0)_12_. The residuals were near normally distributed.

*Bayes Factors*

Bayes factors were derived using an online calculator for the final best fitting adjusted model (17) to disentangle whether there was evidence for the null hypothesis of no effect (Bayes Factor <1/3), insensitive data (Bayes Factor between 1/3 and 3) or evidence for the alternative hypothesis of an effect (Bayes Factor greater than 3). A half-normal distribution was assumed with a percentage change in the prevalence of quit success of 0.1% based on what has been previously detectable using ARIMA type models (18).

**Supplementary Table 1:** Descriptive statistics of the time series of prevalence of successful quit attempts over time and the predictor time series of interest

|  | Mean | SD | 95% confidence interval | |
| --- | --- | --- | --- | --- |
|  |  |  | Lower | Higher |
| Mass media spend (£ millions) | 0.40 | 0.38 | 0.34 | 0.46 |
| E-cigarette use during a quit attempt | 16.60 | 15.55 | 14.03 | 19.17 |

Note: SR=Smoking Reduction; NRT=Nicotine Replacement Therapy; OTC=over the counter

**Supplementary Table 2:** Results of the unadjusted ARIMAX models assessing the association between the variables of interest and prevalence of successful attempts to quit smoking

|  | B  No seasonal AR  Seasonal AR | Lower CI | Upper CI | P | Β  No seasonal AR  Seasonal AR |  |
| --- | --- | --- | --- | --- | --- | --- |
| Mass media spend (£ millions) | 0.042  0.043 | -0.007  -0.007 | 0.091  0.093 | 0.098  0.093 | 0.152  0.157 | ARIMAX(0,1,1)  No lag |
| E-cigarette use during a quit attempt | **0.063**  **0.063** | **0.037**  **0.037** | **0.089**  **0.090** | **<0.001**  **<0.001** | **0.376**  **0.377** | ARIMAX(0,1,1)  No lag |

**Supplementary Table 3:** Results of the adjusted ARIMAX models assessing the association between the variables of interest and prevalence of successful quit attempts

|  | B  No seasonal AR  Seasonal AR | Lower CI | Upper CI | P | β  No seasonal AR  Seasonal AR | Bayes factors |
| --- | --- | --- | --- | --- | --- | --- |
| Partial point-of-sale ban | 0.124 | -0.360 | 0.608 | 0.616 |  | 1.09 |
|  | 0.125 | -0.361 | 0.610 | 0.614 |  | 1.09 |
| Smokefree | 0.178 | -0.310 | 0.667 | 0.474 |  | 1.17 |
|  | 0.179 | -0.310 | 0.669 | 0.473 |  | 1.17 |
| Increase in age of sale (1 month lag) | 0.442 | -0.053 | 0.937 | 0.080 |  | 1.70 |
|  | 0.440 | -0.058 | 0.938 | 0.083 |  | 1.68 |
| Pictorial health warnings | -0.140 | -0.616 | 0.337 | 0.566 |  | 1.11 |
|  | -0.139 | -0.616 | 0.337 | 0.567 |  | 1.11 |
| Move of Stop Smoking Services to local authority control | 0.210 | -0.279 | 0.698 | 0.400 |  | 1.22 |
|  | 0.208 | -0.286 | 0.701 | 0.409 |  | 1.21 |
| Licensing of NRT for harm reduction (1 month lag) | **0.635** | **0.068** | **1.201** | **0.028** |  | **1.87** |
|  | **0.636** | **0.068** | **1.203** | **0.028** |  | **1.87** |
| NICE guidance on harm reduction | 0.217 | -0.292 | 0.726 | 0.403 |  | 1.21 |
|  | 0.214 | -0.301 | 0.730 | 0.415 |  | 1.20 |
| E-cigarette use for SR | -0.041 | -0.112 | 0.029 | 0.252 | 0.036 | 1.03 |
|  | -0.041 | -0.112 | 0.030 | 0.253 | 0.036 | 1.03 |
| Mass media spend | **0.050** | **0.005** | **0.096** | **0.029** | **0.051** | **4.01** |
|  | **0.050** | **0.004** | **0.096** | **0.034** | **0.050** | **3.85** |
| NRT use for SR | -0.174 | -0.384 | 0.037 | 0.105 | 0.036 | 2.33 |
|  | -0.174 | -0.386 | 0.037 | 0.106 | 0.036 | 2.31 |
| Roll-your-own smokers | 0.559 | -0.031 | 1.149 | 0.063 | 0.084 | 1.63 |
|  | 0.562 | -0.037 | 1.161 | 0.066 | 0.085 | 1.61 |
| Non-daily smokers | -0.015 | -0.223 | 0.192 | 0.887 | -0.007 | 0.79 |
|  | -0.018 | -0.242 | 0.207 | 0.877 | -0.008 | 0.82 |
| Smokers’ expenditure on smoking | 0.107 | -0.939 | 1.153 | 0.841 | 0.007 | 1.01 |
|  | 0.104 | -0.946 | 1.154 | 0.846 | 0.007 | 1.01 |
| Cigarettes per day | 0.073 | -0.992 | 1.138 | 0.893 | 0.012 | 1.00 |
|  | 0.074 | -0.992 | 1.140 | 0.892 | 0.013 | 1.01 |
| Smokers | 0.545 | -0.109 | 1.198 | 0.102 | 0.067 | 1.46 |
|  | 0.545 | -0.109 | 1.198 | 0.102 | 0.067 | 1.46 |
| High motivation to quit | 0.031 | -0.277 | 0.339 | 0.843 | <0.001 | 0.92 |
|  | 0.030 | -0.278 | 0.339 | 0.846 | <0.001 | 0.92 |
| Age | 0.561 | -1.882 | 3.004 | 0.653 | 0.009 | 1.03 |
|  | 0.571 | -1.893 | 3.035 | 0.650 | 0.009 | 1.03 |
| Lower social grade | -0.220 | -1.205 | 0.765 | 0.661 | -0.008 | 1.05 |
|  | -0.225 | -1.225 | 0.775 | 0.659 | -0.009 | 1.05 |
| E-cigarette use during a quit attempt | **0.107** | **0.010** | **0.203** | **0.030** | **0.125** | **5.76** |
|  | **0.107** | **0.010** | **0.203** | **0.030** | **0.125** | **5.76** |
| Prescription medication use during a quit attempt | **0.137** | **0.002** | **0.272** | **0.046** | **0.077** | **4.12** |
|  | **0.137** | **0.002** | **0.272** | **0.047** | **0.077** | **4.12** |
| NRT use OTC during a quit attempt | 0.108 | -0.126 | 0.341 | 0.367 | 0.011 | 1.31 |
|  | 0.109 | -0.130 | 0.348 | 0.371 | 0.011 | 1.30 |
| Face-to-face behavioural support during a quit attempt | 0.039 | -0.034 | 0.112 | 0.293 | 0.040 | 0.95 |
|  | 0.040 | -0.035 | 0.114 | 0.297 | 0.040 | 0.97 |
| Quit attempts | -0.152 | -0.574 | 0.270 | 0.480 | -0.023 | 1.17 |
|  | -0.149 | -0.582 | 0.283 | 0.498 | -0.023 | 1.16 |

Note: SR=Smoking Reduction; NRT=Nicotine Replacement Therapy; OTC=over the counter

**References**

1. R Development Core Team. R: A language and environment for statistical computing. R foundation for statistical computing, Vienna, Austria. 2008 [Available from: <http://www.R-project.org>.

2. Fidler JA, Shahab L, West O, Jarvis MJ, McEwen A, Stapleton JA, et al. 'The smoking toolkit study': a national study of smoking and smoking cessation in England. BMC Public Health. 2011;11(1):479.

3. Moritz S, Bartz-Beielstein T. imputeTS: time series missing value imputation in R. The R Journal. 2017;9(1):207-18.

4. Bishop G, Welch G. An introduction to the Kalman filter. Proc of SIGGRAPH, Course. 2001;8(27599-3175):59.

5. Harvey AC. Forecasting, structural time series models and the Kalman filter: Cambridge university press; 1990.

6. Box GE, Jenkins GM, Reinsel GC. Time series analysis: forecasting and control: John Wiley & Sons; 2011.

7. Wakefield MA, Coomber K, Durkin SJ, Scollo M, Bayly M, Spittal MJ, et al. Time series analysis of the impact of tobacco control policies on smoking prevalence among Australian adults, 2001? 2011. Bulletin of the World Health Organization. 2014;92(6):413-22.

8. Cryer JD, Chan K-S. Time series analysis - with applications in R. London: Springer-Verlag New York; 2008.

9. Box GE, Tiao GC. Intervention analysis with applications to economic and environmental problems. Journal of the American Statistical association. 1975;70(349):70-9.

10. López-de-Lacalle J. tsoutliers R Package for Detection of Outliers in time Series. CRAN, R​​ Package. 2016.

11. Munro BH. Statistical methods for health care research: lippincott williams & wilkins; 2005.

12. Lee D, Schmidt P. On the power of the KPSS test of stationarity against fractionally-integrated alternatives. Journal of econometrics. 1996;73(1):285-302.

13. Osborn DR, Chui AP, Smith JP, Birchenhall CR. Seasonality and the order of integration for consumption*. Oxford Bulletin of Economics and Statistics. 1988;50(4):361-77.

14. Yaffee R. An Introduction to Forecasting Time Series with Stata. Taylor & Francis; 2012.

15. Yaffee RA, McGee M. An introduction to time series analysis and forecasting: with applications of SAS® and SPSS®: Academic Press; 2000.

16. Montgomery DC, Jennings CL, Kulahci M. Introduction to time series analysis and forecasting: John Wiley & Sons; 2015.

17. Dienes Z. Using Bayes to get the most out of non-significant results. Frontiers in psychology. 2014;5.

18. Beard E, Bruguera C, Brown J, McNeill A, West R. Was the expansion of the marketing license for nicotine replacement therapy in the United kingdom to include smoking reduction associated with changes in use and incidence of quit attempts? Nicotine Tob Res. 2013;15(10):1777-81.
